# Low-cost Lateral Canthotomy and Cantholysis Model for the Emergency Department

**DOI:** 10.18502/jovr.v19i1.15450

**Published:** 2024-03-14

**Authors:** David McMaster, Anas Khan

**Affiliations:** ^1^Imperial College London, London, UK; ^2^Imperial College Healthcare NHS Trust, London, UK; ^6^These two authors contributed equally to this work.

##  PRESENTATION

Orbital compartment syndrome is a sight-threatening ophthalmic emergency requiring urgent management with lateral canthotomy and cantholysis.^[1]^ Acute retrobulbar hemorrhage secondary to trauma is the most common cause of orbital compartment syndrome, and cases may present out-of-hours to the Emergency Department (ED) without immediate presence of ophthalmologists.^[2]^ Recognizing and acutely managing orbital compartment syndrome is therefore essential when working in Emergency Medicine. A 2018 survey of ED doctors in the UK identified only 37% would perform lateral canthotomy and cantholysis themselves, with 91% indicating this was due to lack of training.^[3]^ Confidence amongst UK ophthalmic trainees is also low, citing limited exposure and limited simulation, with only 23% of regions offering structured simulation activity for lateral canthotomy.^[4]^ Obtaining and maintaining competency with procedural skills can be challenging with lack of exposure, and regular simulated training is essential. Several low-cost eye models for simulating lateral canthotomy and cantholysis have been proposed, and we aimed to develop and validate a model using materials readily available in the ED.^[5]^

**Figure 1:**
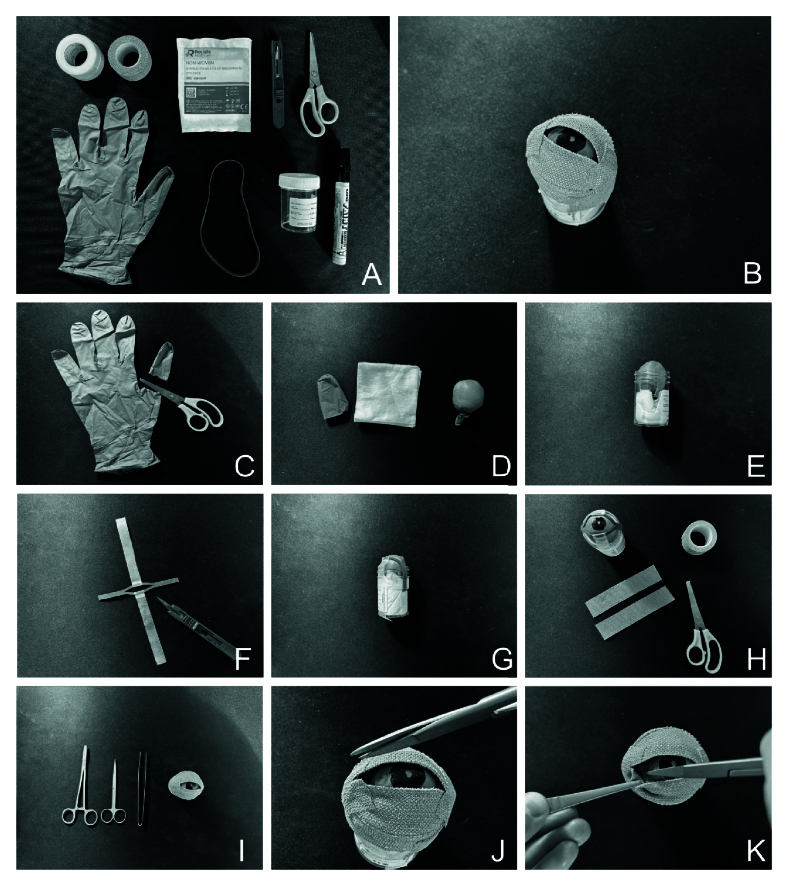
Lateral canthotomy and cantholysis model: Equipment required (A). Model eye (B). Montage of images for assembly of model eye (C–H). Model in use (I–K).

A low-cost model was developed with quick assembly [Figure 1] and readily available materials (i.e., rubber glove, universal container, gauze, rubber band, tape, forceps, and scissors). Eleven junior doctors (ranging from FY2 to ST2) working at an ED in London completed pre- and post-simulated session questionnaires (Qualtrics XM surveys) using a five-point Likert scale (1 = not at all confident, 2 = slightly confident, 3 = somewhat confident, 4 = quite confident, 5 = extremely confident). All participants agreed to take part in the study through informed consent. Statistical analysis was performed using R 3.4.3 software.

##  DISCUSSION

Participants reported a significant increase in confidence when preparing the equipment to perform a lateral canthotomy (pre-session median:1, IQR:0; post-session median:4, IQR:1, *P* < 0.001; Wilcoxon signed-rank test) and with performing a lateral canthotomy and cantholysis (pre-session median:1, IQR:0; post-session median:4, IQR:2, *P* < 0.001; Wilcoxon signed-rank test). In addition, 100% of participants strongly agreed that use of simulated model eyes improved their understanding of how to perform a lateral canthotomy. Overall, trainees felt the simulated model provided enough anatomical detail to understand and perform the steps of the procedure and was appropriate for their level of training.

Irreversible vision loss can occur rapidly with orbital compartment syndrome and trainees involved in acute care are expected to develop and maintain a skillset that allows them to competently manage this ophthalmic emergency. We show that a reproducible low-cost model made with readily available equipment can be used to create simulated conditions to practice basic steps in lateral canthotomy and cantholysis, and improve trainee confidence.

##  Financial Support and Sponsorship

None.

##  Conflicts of Interest 

None.
